# FGF-23: More Than a Regulator of Renal Phosphate Handling?

**DOI:** 10.1002/jbmr.170

**Published:** 2010-06-30

**Authors:** Harald Jüppner, Myles Wolf, Isidro B Salusky

**Affiliations:** 1 Endocrine Unit and Pediatric Nephrology Unit, Massachusetts General Hospital Boston, MA, USA; 2 Division of Nephrology and Hypertension, Department of Medicine, University of Miami Miller School of Medicine Miami, FL, USA; 3 Division of Pediatric Nephrology, Department of Pediatrics, David Geffen School of Medicine, University of California Los Angeles Los Angeles, CA, USA

**Keywords:** PTH, 1,25(OH)_2_D_3_, FGF-23, phosphate homeostasis, chronic kidney disease, bone mineralization, cardiovascular disease

## Abstract

Fibroblast growth factor 23 (FGF-23) is likely to be the most important regulator of phosphate homeostasis, which mediates its functions through FGF receptors and the coreceptor Klotho. Besides reducing expression of the sodium-phosphate cotransporters NPT2a and NPT2c in the proximal tubules, FGF-23 inhibits the renal 1α-hydroxylase and stimulates the 24-hydroxylase, and it appears to reduce parathyroid hormone (PTH) secretion in short-term studies. FGF-23 synthesis and secretion by osteocytes and osteoblasts is upregulated through 1,25-dihydroxyvitamin D_3_ [1,25(OH)_2_D_3_] and through an increased dietary phosphate intake. FGF-23 levels are elevated or inappropriately normal in patients with tumor-induced osteomalacia and several inherited hypophosphatemic disorders, but the most significant increases are found in patients with chronic kidney disease (CKD). During the early stages of CKD, increased FGF-23 production enhances urinary phosphate excretion and thus prevents the development of hyperphosphatemia, reduces the circulating levels of 1,25(OH)_2_D_3_, and therefore contributes to the development of secondary hyperparathyroidism. In patients with end-stage renal disease (ESRD), FGF-23 levels can be extremely high and were shown to be predictors of bone mineralization, left ventricular hypertrophy, vascular calcification, and mortality. It remains to be determined, however, whether FGF-23 represents simply a sensitive biomarker of an abnormal phosphate homeostasis or has, independent of serum phosphate levels, potentially negative “off-target” effects. Nonetheless, reducing the production and/or the biologic activity of FGF-23 may be an important therapeutic goal for this patient population. © 2010 American Society for Bone and Mineral Research.

## Introduction

Parathyroid hormone (PTH) and active vitamin D metabolites are the most important regulators of calcium homeostasis, and much knowledge regarding their mode of actions has accumulated over several decades.(1–3) PTH also affects phosphate homeostasis through its actions on kidney and bone, whereas 1,25-dihydroxyvitamin D_3_ [1,25(OH)_2_D_3_], which is generated from the precursor 25-hydroxyvitamin D through the renal 1α-hydroxylase, contributes to intestinal phosphate absorption.(3) However, both hormones are not the only and probably not the most important hormonal regulators of phosphate homeostasis. Considerable advances that contributed to a much improved understanding of the regulation of phosphate homeostasis were made over the last decade through the molecular definition of rare inherited disorders characterized by either hypo- or hyperphosphatemia and through studies of rare tumors that cause urinary phosphate wasting (for review, see refs. 4 through 7). These efforts have resulted in the discovery of fibroblast growth factor 23 (FGF-23), which is likely the most important phosphate-regulating hormone.(8,9) Furthermore, several other genes were identified that contribute importantly to the regulation of FGF-23 synthesis and secretion, or facilitate high-affinity receptor binding and signal transduction in target cells. It is almost certain, however, that additional genes exist that contribute importantly to these regulatory processes.

Besides its important role in regulating renal phosphate excretion and thus serum phosphate concentration in healthy individuals, there is growing evidence to suggest that FGF-23 has an important role in maintaining normal blood phosphate levels during the early stages of chronic kidney disease (CKD) by augmenting per-nephron urinary phosphate excretion. Moreover, elevated FGF-23 levels were found to predict rapid progression of kidney disease, and among patients with end-stage renal disease (ESRD) treated with dialysis, FGF-23 levels were associated with abnormal bone metabolism.(10) left ventricular hypertrophy and mass index,(11) vascular calcifications,(12) and increased mortality.(13,14) FGF-23 thus appears to have an important role as a biomarker in CKD patients, and measuring FGF-23 levels is likely to provide a sensitive diagnostic tool for assessing phosphate homeostasis and possibly its detrimental “off-target” effects. FGF-23 may thus constitute an important target for therapeutic interventions in the different stages of CKD.

## FGF-23: An Important Regulator of Phosphate Homeostasis

FGF-23 was first identified in mouse embryonic tissue by homology-based hybridization techniques.(15) However, its important role in phosphate homeostasis became apparent only when gain-of-function mutations in *FGF23* were shown to cause autosomal dominant hypophosphatemic rickets (ADHR)(8) and when FGF-23, independently isolated from tumors that cause hypophosphatemia and consequently osteomalacia owing to renal phosphate wasting [tumor-induced osteomalacia (TIO)], was shown to increase renal phosphate excretion.(9) The human *FGF23* gene on chromosome 12p13.3 encodes a glycoprotein consisting of 251 amino acid residues, including a leader sequence of 24 residues that is cleaved off before secretion into the circulation. FGF-23 undergoes *O*-glycosylation by UDP-*N*-acetyl-α-d-galactosamine:polypeptide *N*-acetylgalactosaminyl-transferase 3 (GALNT3), most likely involving amino acid residue 178.(16) This posttranslational modification protects FGF-23 from cleavage by subtilisin-like proprotein convertases.(17) Consequently, inactivating mutations in *GALNT3* or *FGF23* that prevent normal *O*-glycosylation of FGF-23 leading to the synthesis of an unmodified molecule,(18) which results in impaired secretion of intact but not C-terminal FGF-23.(18–21) *O*-glycosylation is thus required for secretion of intact, biologically active FGF-23.(21)

**Fig. 1:**
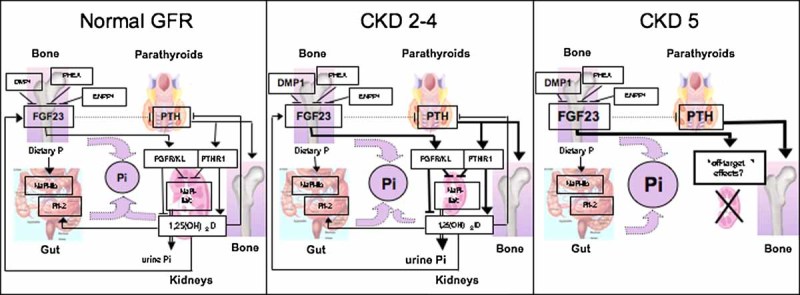
Regulation of phosphate homeostasis in health and chronic kidney disease (CKD). (*A*) FGF-23, one of the most important regulators of phosphate homeostasis, is produced by osteocytes and osteoblasts in response to 1,25(OH)_2_D_3_ and when the dietary intake of phosphate is increased. PHEX, DMP1, and ENPP1 are most likely upstream regulators of FGF23 synthesis because inherited human disorders that are caused by a lack of these proteins are associated with increased FGF-23 levels and increased urinary phosphate excretion. Phosphate is absorbed in the gut from the diet, stored in the skeleton, and excreted by the kidneys. The sodium-dependent phosphate cotransporter NPT2b contributes significantly to the absorption of dietary phosphate, which is enhanced by 1,25(OH)_2_D_3_. FGF-23 activates different FGF receptors when associated with the coreceptor Klotho and it increases renal phosphate clearance by reducing expression of the sodium-dependent phosphate cotransporters NPT2a and NPT2c in the proximal renal tubules. It furthermore suppresses the renal 1α-hydroxylase and stimulates the 24-hydroxylase activity (not shown) and thus reduces 1,25(OH)_2_D_3_ levels through two different mechanisms. FGF-23 also may act on the parathyroid glands to decrease PTH production (*dashed line*). PTH activates the PTH/PTHrP receptor (PTHR1) and thereby increases renal phosphate clearance and 1,25(OH)_2_D_3_ synthesis. (*Modified with permission from ref*. (77).) (*B*) As renal function declines (CKD stages 2 to 4), FGF-23 levels increase, thereby enhancing renal phosphate clearance to help maintain serum phosphate levels within the normal range. Elevated FGF-23 levels, however, reduce 1,25(OH)_2_D_3_ levels and thus contribute to the development of secondary hyperparathyroidism. (*C*) In patients with end-stage renal disease (CKD stage 5) treated by dialysis, FGF-23 levels can be dramatically elevated and thus may have “off-target” effects in different tissues.

Osteocytes and osteoblasts are the main source of FGF-23,(22–24) but low levels of uncertain significance are also detected at the transcriptional level in the ventrolateral thalamic nucleus, the thymus, the small intestine, and the heart.(25) Several proteins appear to suppress the synthesis and secretion of FGF-23, including phosphate-regulating protein with homology to endopeptidases on the X chromosome (PHEX), dentin matrix protein 1 (DMP1), and ectonucleotide pyrophosphatase/phosphodiesterase 1 (ENPP1)(4–7,26,27) (Fig. 1, *left panel*). The most important stimulators of FGF-23 synthesis appear to be dietary phosphate(28–30) and 1,25(OH)_2_D_3_,(31) but other agents, such as some iron formulations, also may increase its production.(32–34) However, owing to the lack of suitable cell lines, it so far has been impossible to study in detail the regulation of FGF-23 synthesis and secretion and the impact of different pharmacological agents.

FGF-23 mediates its phosphate-regulating properties through different FGF receptors (FGFRs), most prominently through FGFR1c; these actions require α-Klotho (KL) as coreceptor.(35,36) However, the site(s) of FGF-23 action in the kidney remains controversial. FGF-23 decreases expression of NPT2a and NPT2c,(37,38) it decreases the activity of the 1α-hydroxylase, and it increases the activity of the 24-hydroxylase in the proximal tubules.(6,39) However, the coreceptor Klotho, which is essential for mediating the phosphaturic actions of FGF-23, is expressed mainly in the distal tubules. In this portion of the kidney, the injection of FGF-23 rapidly induces phosphorylation of MAPK and expression of the early growth-response gene 1 (Egr-1) which is accompanied by a reduction in Npt2a expression in adjacent proximal tubules.(40) These findings could indicate either that FGF-23 uses a different signaling pathway in the proximal tubules or that it induces the secretion of an “intermediary phosphatonin” from the distal tubules that affects Npt2a (and possibly Npt2c expression) in the proximal tubules through paracrine mechanisms. Alternatively, low levels of KL expression in the proximal renal tubules may be sufficient for mediating the phosphaturic actions of FGF23.

## Assays to Measure FGF-23

Serum FGF-23 levels can be measured routinely with immunometric assays that detect either the intact hormone alone (iFGF-23 assay)(41,42) or with assays that detect the intact FGF-23 as well as C-terminal fragments thereof (cFGF-23 assay).(43) One of the currently available iFGF-23 assays provides better sensitivity than the cFGF-23 assay to establish the diagnosis of FGF-23-dependent hypophosphatemic disorders.(42) In patients with ESRD, however, who usually have elevated FGF-23 levels, results obtained with the different assay systems are well correlated and provide an excellent prediction of FGF-23 bioactivity.(10,13,44) Furthermore, Western blot analysis of sera from dialysis patients has revealed that circulating FGF-23 comprises predominantly the intact hormone,(44) suggesting that either of the currently available immunoassays accurately assesses bioactive FGF-23 and thus is suitable for exploring the pathophysiologic roles of this novel hormone in patients with impaired renal function.

## Increased FGF-23 Bioactivity Enhances Urinary Phosphate Excretion in Individuals with Normal Renal Function

Unlike most other fibroblast growth factors, FGF-23 is readily detected in the circulation of healthy individuals,(41,43,45) and serum levels rise in humans and rodents in response to phosphate loading.(28–30) These findings make it likely that FGF-23 is an important regulator of renal phosphate handling in individuals with normal renal function.

Increased FGF-23 bioactivity is observed in several inherited or acquired disorders. These include ADHR, a rare inherited disorder that is caused by heterozygous *FGF23* missense mutations involving amino acid residue 176 or 179, which constitute the recognition/cleavage site for subtilisin-like proprotein convertases; these mutations thus impair intracellular cleavage of full-length FGF-23 but do not routinely cause significant elevations in circulating FGF-23 levels.(46) Serum FGF-23 levels are elevated in patients with TIO, an acquired disorder that is caused by usually small, often difficult-to-locate tumors that express abundant amounts of *FGF23* mRNA and protein. These patients often show major elevations in serum FGF-23 levels and consequently severe hypophosphatemia owing to increased urinary phosphate excretion and decreased 1,25(OH)_2_D_3_ levels.(41–43,47) Other inherited disorders with increased or inappropriately normal FGF-23 levels include X-linked hypophosphatemia (XLH), which is caused by inactivating mutations in *PHEX*,(41,43,45) and two forms of autosomal recessive hypophosphatemia (ARHP) that are caused by homozygous mutations in either *DMP1*(48,49) or *ENPP1.*(26,27) Other rare disorders that are associated with increased serum FGF-23 levels include fibrous dysplasia (caused by heterozygous *GNAS* mutations that lead to the formation of a constitutively active Gs-alpha),(50) Jansen's metaphyseal chondrodysplaia (caused by heterozygous activating *PTHR1* mutations),(51) osteoglophonic dysplasia (caused by heterozygous activating *FGFR1* mutations),(52) and the linear nevus sebaceous syndrome (LNSS; molecular defect unknown).(53,54)

## FGF-23 Enhances Phosphate Excretion in Early CKD Stages

Circulating FGF-23 levels are already elevated in patients with CKD stages 2 and 3, that is, well before there is a critical reduction in functioning nephrons [CKD stages 1 and 2: glomerular filtration rate (GFR) > 60 mL/min per 1.73 m^2^; stage 3: 30 to 59 mL/min per 1.73m^2^, stage 4: 15 to 29 mL/min per 1.73m^2^; stage 5: <15 mL/min per 1.73m^2^]. This rise in serum FGF-23 concentration appears to be a reflection of an increased production by osteocytes rather than accumulation because of impaired renal function, and it precedes any detectable increase in serum phosphate concentration and the decline in serum 1,25(OH)_2_D_3_ levels.(24,55,56) In fact, increased FGF-23 levels in these early CKD stages are associated with increased fractional excretion of phosphate, making it likely that enhanced FGF-23 secretion helps to maintain normophosphatemia despite a reduction in nephron mass (Fig. 1, *middle panel*). Although FGF-23 measurements may provide a sensitive biomarker of abnormal renal phosphate handling in early CKD, it remains unknown how the osteocyte “senses” subtle abnormalities in renal tubular phosphate handling and whether a kidney-specific factor exists that triggers the release of FGF-23 from bone cells in response to transient elevations in serum phosphorus level or in response to unspecified disease processes in the kidney.

The elevation in serum FGF-23 levels in CKD stages 2 and 3 most likely precedes the increase in PTH levels, thus raising the possibility that secondary hyperparathyroidism develops, at least in part, because of an FGF-23-induced reduction in the renal production of 1,25(OH)_2_D_3_, an important negative regulator of PTH synthesis.(55,56) On the other hand, FGF-23 was shown in short-term studies to directly reduce the secretion of PTH in vitro and in vivo,(57,58) suggesting that FGF-23 has two competing effects on PTH synthesis and/or secretion—an indirect effect through an FGF-23-dependent reduction in 1,25(OH)_2_D_3_ synthesis and a direct effect on parathyroid cells, which express different FGF receptors as well as Klotho and thus can bind FGF-23 with high affinity. However, the expression levels of Klotho and FGFRs are reduced in parathyroid tissue of patients with secondary hyperparathyroidism, which could explain why FGF-23, even if present at extremely high levels, cannot prevent a rise in PTH levels.(59)

## FGF-23 Predicts Bone Mineralization in CKD Stage 5

Abnormalities in PTH, calcium, phosphate, and vitamin D explain some, but not all, of the complex alterations in bone structure and function that accompany CKD. Traditionally, PTH measurements represented the only hormonal biomarker to predict bone turnover in ESRD patients, and thus they are used to guide therapy with active vitamin D analogues, calcimimetics, and phosphate binders. To acknowledge that renal osteodystrophy affects not only bone turnover, Kidney Disease: Improving Global Outcomes (KDIGO) introduced a new classification that also includes bone mineralization and volume (TMV).(60)

FGF-23 levels were shown recently to correlate with parameters of altered skeletal mineralization in children with CKD.(10,24) Although the mechanisms leading to defective skeletal mineralization in CKD patients remain to be evaluated, FGF-23 levels were inversely correlated with osteoid surface, osteoid volume, and osteoid thickness across the spectrum of CKD.(10) FGF-23 is produced by osteocytes and osteoblasts, and its expression in bone from patients with CKD, who were naive to vitamin D therapy, was shown to be markedly elevated; this increase in immunoreactive FGF-23 occurs, before vitamin D therapy, very early in the course of CKD, and the amount of FGF-23 protein in bone cells, particularly in osteocytes, increases progressively as GFR diminishes.(24) It is interesting to note that the expression of DMP1, inactivating mutations in which lead to increased FGF-23 expression,(48,49) is also markedly elevated, yet its expression pattern differs from that of FGF-23.(24) These findings provide further evidence for a potential role of bone in responding to phosphate load by increasing FGF-23 production early in the course of CKD. In patients treated with dialysis, FGF-23 thus appears to have a significant role as a biomarker that predicts some aspects of the abnormalities of bone metabolism that are observed in this condition and are part of the spectrum of metabolic bone disease (MBD) associated with CKD.(60)

## Postrenal Transplantation Hypophosphatemia Is Triggered by FGF-23

Successful renal transplantation frequently corrects many of the abnormalities in mineral and bone metabolism that develop during the course of CKD, but hyperparathyroidism frequently persists and was thought to contribute to the development of hypercalcemia and hypophosphatemia in the extended posttransplant period. More recently, however, persistently elevated FGF-23 levels were observed for prolonged periods of time after renal transplantation, and these have been associated with hypophosphatemia and low 1,25(OH)_2_D_3_ level independent of PTH.(61,62) It is plausible that patients who received a renal transplant and show persistently increased FGF-23 secretion despite a well-functioning kidney graft are at risk of accelerated deterioration of renal function just as nondiabetic CKD patients without a prior renal transplant(63); this is an important question that requires further study. Aside from its effects on mineral ion homeostasis, FGF-23 levels therefore may help to predict long-term graft function. Future outcomes studies in transplant populations are needed to test this hypothesis.

## Plausible “Off-Target” Effects of FGF-23 in ESRD

In patients with early stages of CKD, increased FGF-23 levels reduce 1,25(OH)_2_D_3_ formation and are likely to contribute prominently to maintaining serum phosphate levels within normal limits.(55,56) However, in these patients, FGF-23 also may have systemic “off-target” effects that contribute to cardiovascular disease, a leading cause of death in both adult and pediatric CKD patients. Indeed, several studies involving CKD patients have suggested recently a pathogenic role of FGF-23 in the development of cardiovascular injury because high FGF-23 levels were found to be associated with vascular calcifications, increased left ventricular mass index, and left ventricular hypertrophy in dialysis patients, predialysis patients, and subjects with normal renal function.(12,64,65) Furthermore, FGF-23 levels were independently associated with increased mortality risk in incident dialysis patients without confounding by serum phosphate levels or a variety of other factors that have been associated with dialysis mortality previously.(13) Similarly dramatic findings of a dose-response type of relationship between FGF-23 and mortality were made in additional studies investigating long-term dialysis patients.(14) In each of these studies, the impact of serum FGF-23 concentration was independent of serum phosphate levels, suggesting that either FGF-23 is superior to serum phosphate as a biomarker of phosphate-related toxicity or an increased FGF-23 level by itself has negative biologic consequences. The clinical implications of increased FGF-23 values in dialysis patients remain to be established because therapy with active vitamin D sterols further increase FGF-23 levels but, on the other hand, is associated with a survival benefit that is independent of serum calcium, phosphate, and PTH levels in dialysis patients.(66–68) This paradox highlights the needs for future prospective, randomized trials to evaluate the impact of vitamin D sterol therapy and FGF-23 levels in patients treated with maintenance dialysis. Identification of vitamin D analogues that have untainted survival benefits yet limited effects on the production of FGF-23 could provide a considerable therapeutic benefit.

## Therapeutic Interventions to Limit the Putative “Off-Target” Effects of FGF-23 in CKD

As outlined earlier, growing evidence indicates that measurement of FGF-23 may serve as a biomarker that provides evidence for a disordered mineral ion homeostasis in CKD patients and predicts abnormal bone mineralization, kidney disease progression, vascular calcifications, and overall mortality. However, interpretation of FGF-23 levels depends on the CKD stage and the specific therapeutic interventions based on the degree of renal function. Furthermore, it is conceivable that FGF-23 has direct “off-target” effects that contribute to some of the observed morbidity and mortality in this patient population.

Elevated serum phosphorus is an established independent risk factor for cardiovascular disease and mortality in patients treated with dialysis(69) (as well as in predialysis CKD patients and in individuals without kidney disease; for review, see refs. (64) and (65)). Consistent with the importance of elevated serum phosphorate levels as predictors of poor outcome, early treatment of incident hemodialysis patients with phosphate binders was shown to be associated with a significantly lower mortality risk.(64) Interestingly, the survival benefit of early binder therapy was independent of serum phosphate levels, suggesting that other factors such as FGF-23 could contribute to mortality through yet unknown mechanisms. This then raises the question of whether treatment with phosphate-binding agents could improve outcome by lowering the FGF-23 levels in patients with earlier and later stages of CKD, as suggested recently,(70–72) and whether additional treatment options could be envisioned that reduce potential “off-target” effects of FGF-23.

## Phosphate Binders in Early Stages of CKD?

Currently, therapy with phosphate binders is recommended to correct hyperphosphatemia; prospective, randomized long-term trials need to be conducted in patients with CKD stages 2 to 4 to evaluate whether phosphate binders could lead to a sustained reductions in serum FGF-23 levels and whether such reductions would prolong kidney and patient survival and prevent the development of secondary hyperparathyroidism, as predicted by earlier studies. As a first step, it must be determined whether phosphate binders effectively lower FGF-23 in this patient population. A recent pilot study of 40 patients with stage 3 to 4 CKD and normal serum phosphate levels demonstrated that sevelamer, but not calcium-based binders, lowered FGF-23 over a 6-week treatment period despite comparable reductions in urinary phosphate across the two treatment arms.(72) These findings confirmed previous reports in animals(71) and a similar small study in dialysis patients.(70) Additional studies with longer follow-up times are needed to confirm this initial report in stages 2 to 4 and to determine whether the lack of an FGF-23-lowering effect of calcium was an outlying finding in a small study or represents a true physiologic difference compared with the non-calcium-based binders.

In addition to establishing what regimens can best lower FGF-23 for sustained periods in stages 2 to 4 CKD, it must be demonstrated that FGF-23 excess represents a risk factor for adverse outcomes in this population, as suggested by several investigators. Once additional studies have provided further evidence supporting the conclusion that FGF-23 is a risk factor for mortality in different CKD stages, and if FGF-23 can be lowered effectively with sustainable medical regimens, large clinical trials should be conducted. The role of dietary phosphate restriction and possibly niacin administration (which lowers NPT2b expression and thus gastrointestinal phosphate absorption(73)) in such trial also requires significant attention either as comparator arms or, more likely, as complementary therapies in a multi-intervention approach to be compared with placebo.

## Monoclonal Anti-FGF-23 Antibodies as Inhibitors of FGF-23 Action in CKD

Therapy with monoclonal anti-FGF-23 antibodies were shown recently in *hyp* mice, that is, the murine equivalent of *XLH*, to reduce FGF-23 bioactivity, thereby increasing expression of the sodium-dependent phosphate cotransporter in the proximal renal tubules and normalizing serum phosphate levels.(74) Such therapy would not be predicted to be helpful in CKD patients stages 2 to 4; in fact, such an intervention would be expected to carry considerable risk since increased FGF-23 levels in patients with early CKD stages are thought to help maintain serum phosphorus levels within normal limits (see above). Therapy with monoclonal anti-FGF-23 antibodies in CKD stages 2 to 4 probably would result in hyperphosphatemia because of reduced urinary phosphate excretion and enhanced 1,25(OH)_2_D_3_-dependent intestinal phosphate absorption. The latter also could lead to a significant increase in serum calcium that could contribute to hypercalcemia, as observed in *FGF23* and *Klotho* null mice.(75,76)

In patients undergoing maintenance dialysis, FGF-23-mediated renal phosphate elimination is no longer the major determinant of serum phosphate levels. Therefore, in contrast to patients with some degree of residual renal function, monoclonal anti-FGF-23 antibodies may be of potential benefit in ESRD patients if evidence for “off-target” effects of FGF-23 can be confirmed through different in vitro and in vivo approaches. Such studies would help to decide whether high FGF-23 levels are directly contributing to the increased mortality observed in dialysis patients, in which case treatment with monoclonal anti-FGF-23 antibodies could be beneficial. There is currently no possibility to allow long-term survival of nephrectomized laboratory animals through dialysis treatment, thus making it impossible to assess the impact of such antibodies on putative “off-target” effects of FGF-23 in the laboratory setting. Therefore, in vitro approaches need to be developed that can serve as surrogates to then be tested in humans.
